# Binational Analysis of Maternal Mortality Between Brazil and Portugal in 2020–2023: A Population-Based Epidemiological Study

**DOI:** 10.3390/nursrep16060185

**Published:** 2026-05-28

**Authors:** Gustavo Gonçalves dos Santos, Mónica Alexandra Pinho da Silva, Maria João Jacinto Guerra, Júlia Maria das Neves Carvalho, Ana Cristina Ribeiro da Fonseca Dias, Maria Luísa Santos Bettencourt, Cely de Oliveira, Bruna Feichas Renó, Eneida Tramontina Cerqueira, Katucha Rocha de Almeida Farias

**Affiliations:** 1 Programa de Pós-Graduação em Enfermagem, Universidade Guarulhos (UnG), Guarulhos 07023-070, SP, Brazil; 2 Escola Superior de Enfermagem do Porto, Universidade do Porto (ESEP/UP), 4200-072 Porto, Portugal; monicasilva@esenf.pt (M.A.P.d.S.); mjguerra@esenf.pt (M.J.J.G.); 3 Escola Superior de Enfermagem de Coimbra, Universidade de Coimbra (ESEnfC/UC), 3046-851 Coimbra, Portugal; juliacarvalho@ese.uc.pt; 4 Escola Superior de Saúde, Universidade dos Açores (UAc/ESS), 9500-315 Ponta Delgada, Portugal; ana.cr.dias@uac.pt (A.C.R.d.F.D.); maria.ls.bettencourt@uac.pt (M.L.S.B.); 5 Universidade Santa Cecília (UNISANTA), Santos 11045-907, SP, Brazil; celyoliveira@unisanta.br (C.d.O.); brunareno@unisanta.br (B.F.R.); eneidatvc2017@gmail.com (E.T.C.); 6 Universidade Católica de Santos (UNISANTOS), Santos 11070-906, SP, Brazil; katucha.almeida@gmail.com

**Keywords:** mortality, maternal mortality, health inequalities, women’s health, health information system

## Abstract

**Background/Objectives**: Maternal mortality remains an important indicator of health inequities, reflecting social, regional, and racial inequalities, as well as the responsiveness of health systems. This study aimed to analyze and compare maternal mortality between Brazil and Portugal from 2020 to 2023. **Methods**: This is a binational ecological and observational study based on secondary data from official records of live births and maternal deaths in both countries. Maternal mortality rates were calculated per 100,000 live births and stratified by sociodemographic and regional variables. Poisson regression models offset by the logarithm of live births were used to estimate adjusted incidence ratios (IRR) and 95% confidence intervals. Analyses were conducted using R and Stata software. **Results**: Brazil presented rates between 55 and 62 per 100,000 live births, while Portugal maintained lower values, ranging from 8 to 20 per 100,000. In Brazil, higher risks were observed among Black and Indigenous women, residents of the North and Northeast regions, and in age groups above 30 years. Direct and indirect causes showed similar proportions, with an increase in indirect causes during the pandemic. In Portugal, mortality showed low magnitude, but annual fluctuation was attributed to the small number of events and the limitation of microdata. **Conclusions**: The study highlights strong structural and racial inequalities in Brazilian maternal mortality, contrasting with the lower magnitude and greater stability observed in Portugal. This reinforces the need for intersectoral actions, strengthening the obstetric network, and continuous surveillance to reduce preventable deaths and promote equity in maternal care.

## 1. Introduction

Maternal mortality remains a summary indicator of the quality of health systems and social inequalities, reflecting not only acute clinical events associated with pregnancy, childbirth and the postpartum period, but also structural determinants such as access to prenatal care, the structure of referral networks and the socioeconomic characteristics of the populations. In distinct contexts such as Brazil and Portugal, comparative analysis of maternal mortality allows the identification of both clinical factors (obstetric hemorrhage, gestational hypertension, infections and indirect causes) and organizational and equity aspects that influence the risk of maternal death, offering a basis for interventions driven by public policies [1].

In Brazil, despite advances in certain regions and periods, maternal mortality shows signs of instability and marked inequality among population subgroups. Time series analyses and studies on maternal deaths covering up to 2021–2022 recorded punctual increases associated with health crises and highlighted important subnational variations, for example, persistent regional differences and peaks attributable to the impact of the COVID-19 pandemic, in addition to the persistence of direct obstetric causes such as hypertension, hemorrhage, and infection. This evidence indicates that, in Brazil, the sustained reduction in maternal mortality depends both on clinical interventions (better management of obstetric emergencies) and on structural measures that address racial and territorial inequalities [2,3].

Racial and ethnic inequalities emerge as one of the most consistent vectors of vulnerability in the Brazilian context: recent studies show that black women have substantially higher maternal mortality rates than white women, with prevalence ratios that practically double the risk in some analyzed series. This pattern suggests both differential exposure to social determinants of health and possible biases in access to and quality of care during prenatal, childbirth, and postpartum periods, aspects that require investigating specific pathways (e.g., delays in seeking and receiving care, underreporting, and problems in the surveillance of causes of death) [1].

On the other hand, Portugal, like many European countries, presents overall maternal mortality indicators that are much lower than those of Brazil in terms of the ratio per 100,000 live births; however, this does not mean an absence of challenges. Recent Portuguese studies focus on determinants such as maternal health literacy, childbirth experience, organization of care (including childbirth practices and place of birth), and barriers to access for subgroups (immigrants, rural populations), areas that, even in contexts of universal health systems, can generate avoidable risks. Recent literature on Portugal has emphasized the importance of the quality of perinatal care, obstetric safety, and strengthening communication between professionals and pregnant women as critical components to prevent complications that, although rare, can lead to fatal outcomes [4,5].

Comparing Brazil and Portugal requires attention to definitional differences, data sources, and the role of modeled estimates versus administrative records. While national sources (e.g., civil registration systems and mortality databases) provide recorded counts and have allowed detailed analyses in both countries, international bodies and agencies frequently produce modeled estimates to allow comparisons between countries; these estimates can sometimes diverge from national series, especially in years with few absolute events, requiring methodological transparency when interpreting trends and binational comparisons. Thus, comparative studies should explicitly state sources (records vs. estimates), codes used for classifying causes (e.g., O00–O95; O98–O99), and decisions about denominators (live births) to ensure reproducibility [3,6].

Beyond the immediate clinical causes, recent evidence highlights contextual factors that may explain some of the differences between the two countries: organization of maternal health services (capacity for high-risk obstetric care and referral networks), primary care policies and vaccination coverage in pregnant populations (e.g., vaccines that reduce risks from emerging infections), sociodemographic conditions (educational level, poverty), and the presence of care practices that increase risks (e.g., high cesarean section rates in certain contexts). Studies investigating the impacts of the pandemic have shown temporary increases in maternal mortality associated with service overload and specific forms of indirect morbidity, reinforcing that exogenous events exacerbate pre-existing weaknesses in systems [3,7].

From a methodological point of view, the low absolute frequency of maternal deaths in countries with smaller populations or in specific regions makes it essential to use analytical strategies that minimize statistical volatility: multi-year moving averages, aggregated analyses by region or period, and the use of age-standardized measures. Furthermore, the investigation of the causes of maternal death benefits from the combination of quantitative approaches (analysis of records and time series, modeling) with qualitative assessments and maternal death audits when available, which allow the identification of failures in care processes and gaps in surveillance. Compared to binational approaches, integrating these approaches helps to differentiate universal clinical problems from local weaknesses in the health system [8].

Recent literature supports the view that effective interventions to reduce maternal mortality combine clinical actions (protocols for hemorrhage and pre-eclampsia, improved access to emergency obstetric care) with equity policies (reducing barriers to quality prenatal care, programs for vulnerable populations) and continuous monitoring and auditing efforts. In a comparative and translational study between distinct realities, it is possible to identify adaptable good practices, for example, strengthening referral networks, training teams, and communication strategies for at-risk populations that can inform policies in both countries, always considering contextual differences and the need for robust and up-to-date data to track the impact of interventions [3,7,8].

Although Brazil has implemented structuring policies such as the Stork Network (Rede Cegonha), aimed at organizing maternal and child care pathways, improving obstetric care, and reducing inequalities in access, and more recent initiatives such as the Alyne Network (Rede Alyne), focused on racial equity and reducing preventable maternal deaths among Black women, there is still a scarcity of analyses that assess how these models interact with international experiences and which elements are adaptable, transferable, or contrasting in the European context. This gap becomes even more relevant in light of the global goals of the Sustainable Development Goals (SDGs), particularly SDG 3.1, which proposes to significantly reduce maternal mortality by 2030. Both Brazil and Portugal face specific challenges in approaching this goal, whether due to the persistence of internal inequalities (territorial, racial, socioeconomic) in Brazil, or the need to maintain low rates with pressured systems, demographic changes, and variations in access in Portugal. Beyond describing maternal mortality trends, this study contributes to the literature by examining how differences in national health information systems shape the ability to monitor maternal mortality. Cross-national comparisons remain methodologically challenging due to heterogeneity in data availability, granularity, and surveillance capacity. By juxtaposing Brazil and Portugal, this study highlights key challenges and opportunities for the international harmonization of maternal health data. However, this study does not aim to quantify the causal effect of the pandemic, but rather to describe maternal mortality trends during the pandemic period.

This study aims to provide a descriptive comparative analysis of maternal mortality associated with COVID-19 in Brazil and Portugal between 2020 and 2023, exploring temporal trends and sociodemographic inequalities using the best available national data sources in each country. Portugal was selected as a comparator due to its contrasting demographic profile and well-established maternal health surveillance system. Additionally, the study aims to reflect on methodological challenges in cross-country comparisons and the implications for strengthening global maternal mortality surveillance.

## 2. Materials and Methods

### 2.1. Type of Study

This is an analytical population-based observational study reported according to the recommendations of Strengthening the Reporting of Observational Studies in Epidemiology (STROBE). The choice of a population-based design allows for the estimation of national and subnational rates, reduces selection biases, and maximizes the detection of temporal and spatial patterns in rare outcomes such as maternal mortality [9]. Given the ecological and observational design, findings reflect population-level associations and should not be interpreted as causal relationships at the individual level. No formal statistical assessment of the effect of the COVID-19 pandemic (e.g., pre-post comparisons or interaction analyses) was conducted. The pandemic period is considered a temporal context for interpreting trends.

### 2.2. Study Location

The study area encompasses the entire territory of Brazil and Portugal, using both the national level and subnational strata as units of analysis. Brazil and Portugal provide a particularly relevant binational comparison due to their historical, linguistic, and health system connections, while simultaneously presenting markedly different demographic and epidemiological profiles. Brazil is a large middle-income country with continental dimensions and substantial regional and social inequalities, whereas Portugal is a high-income European country with a smaller and more homogeneous population. This contrast offers a valuable opportunity to contextualize maternal mortality patterns across distinct health system structures and sociodemographic contexts. Thus, the Brazil–Portugal comparison enables a contextual and descriptive exploration of maternal mortality trends across contrasting demographic, epidemiological, and health system scenarios.

### 2.3. Study Population

The study population includes all women of reproductive age who are listed in death records as maternal deaths according to the international definition (deaths during pregnancy, childbirth, or up to 42 days after the end of pregnancy due to pregnancy-related causes). For Brazil, microdata from the Sistema de Informação de Mortalidade (SIM) (http://tabnet.datasus.gov.br/cgi/tabcgi.exe?sim/cnv/mat10uf.def) and the Sistema de Informações sobre Nascidos Vivos (SINASC) (http://tabnet.datasus.gov.br/cgi/tabcgi.exe?sinasc/cnv/nvuf.def) were used to extract records classified by codes; for Portugal, datasets from the Instituto Nacional de Estatística (INE) (https://www.ine.pt/xportal/xmain?xpid=INE&xpgid=ine_main) and Pordata from the Francisco Manuel dos Santos Foundation (https://www.pordata.pt/pt) were used, accessed on 20 November 2025.

### 2.4. Data Collection and Study Variables

Data collection was carried out through access to and download of official databases: (i) SIM microdata, and (ii) INE and PORDATA databases (maternal mortality indicators). The Brazilian variables included in the study were extracted directly from the SIM and SINASC microdata, both made available by the Ministry of Health. From these databases, it was possible to characterize in detail the sociodemographic and epidemiological profile of women who died as mothers, allowing the inclusion of essential individual variables for adjusted modeling. For the primary outcome, maternal death, the categories defined according to ICD-10 (O00–O95; O98–O99) were considered, stratified into direct, indirect, or unspecified obstetric maternal death. The explanatory variables included: race/color (white, black, yellow, brown, indigenous, and unknown), age group grouped according to epidemiological patterns (10–14, 15–19, 20–29, 30–39, 40–49, 50–59 years); region of residence (North, Northeast, Southeast, South, and Midwest); education (none, 1–3 years, 4–7 years, 8–11 years, ≥12 years, unknown); and marital status (single, married, widowed, legally separated, other, unknown).

In contrast to the Brazilian scenario, the methodological stage concerning the Portuguese data presented substantial limitations stemming from the way national databases provide information on maternal mortality. The INE and the secondary database PORDATA provide only aggregated indicators, mainly the annual maternal mortality rate (MMR) per 100,000 live births, accompanied by the absolute number of deaths and live births. Unlike Brazil, anonymized microdata that would allow for the stratification of deaths according to individual maternal characteristics, such as race/ethnicity, education, marital status, or age group, are not available. Similarly, it is not possible to classify the causes of death by ICD-10 categories or to differentiate direct and indirect obstetric deaths at the individual level. Consequently, it is not possible to replicate for Portugal the same adjusted multivariate models developed for Brazil, such as Poisson regressions with individual covariates, multilevel models, or interaction assessments. The estimates for Portugal were based on aggregated annual data and the application of time trend models adjusted only for the denominator (live births), acknowledging the greater statistical imprecision resulting from the small number of events and the impossibility of controlling for confounding.

This methodological asymmetry between the database individual microdata in Brazil versus aggregated data in Portugal restricts comparability at more detailed levels, especially regarding the assessment of sociodemographic inequalities. Even so, the integration of the two sources allows for the identification of national trends, differences in the magnitude of maternal mortality between contexts, and potential structural determinants, while respecting the particularities of each country’s information systems. Discussing these limitations is fundamental for the proper interpretation of the results and reinforces the need for greater openness and detail in Portuguese databases, in order to allow for more comprehensive and comparable analyses in international studies.

### 2.5. Data Analysis

Statistical analysis was conducted in descriptive and analytical stages, adapted to the specific data of each country. Initially, descriptive statistics were performed on the sociodemographic and epidemiological variables of Brazilian women who died as mothers, presented in absolute and relative frequencies, according to race/color, age group, region of residence, education level, and marital status (Table 1). Subsequently, deaths were classified according to type (direct, indirect, unspecified), period (pregnancy, childbirth, puerperium), and underlying cause (ICD-10: O00–O99), summarized in Table 2.

To assess the factors associated with maternal mortality, Poisson regression models with an offset for the logarithm of the number of live births were applied, estimating adjusted incidence ratios (IRR) and 95% confidence intervals, considering race, age group, and region of residence as explanatory variables (Table 3).

Maternal mortality ratios were modeled using Poisson regression with robust variance. Model adequacy and assumptions were formally assessed through diagnostic procedures, as detailed below. Overdispersion was assessed by examining the ratio between the deviance and the degrees of freedom, as well as the Pearson chi-square statistics. Values close to 1 indicated no substantial overdispersion. When mild overdispersion was detected, robust standard errors were applied to ensure valid inference. Poisson regression was selected due to the count nature of maternal deaths and the modeling of rates using population offsets. To assess robustness, sensitivity analyses using negative binomial regression were performed. Results were consistent in direction and magnitude, supporting the adequacy of the Poisson specification. Because maternal mortality may cluster geographically, models included regional fixed effects to account for contextual heterogeneity. This approach allowed adjustment for unobserved regional characteristics while preserving model interpretability.

For the Portuguese case, given the unavailability of individual microdata, the analysis was restricted to aggregated annual data on deaths and live births between 2020 and 2023. Annual maternal mortality rates (per 100,000 live births) were estimated, and the temporal trend was modeled using Poisson regression adjusted for the denominator (live births), indicating a non-significant decreasing trend during the period (Table 4). The binational comparison was presented through a time series of maternal mortality rates, showing consistently higher levels in Brazil compared to Portugal, as well as the fluctuation observed in the Portuguese context during the first year of the pandemic. All tests considered a significance level of 5% (*p* < 0.05). The analyses were performed using R software (versions 4.2–4.3; tidyverse, MASS, ggplot2 packages) and Stata 17, ensuring reproducibility and control of model dispersion.

Due to differences in data availability, distinct analytical strategies were required. Brazilian analyses were conducted using individual-level microdata, enabling multivariable modeling and adjustment for sociodemographic variables. In contrast, the Portuguese component relied on nationally aggregated indicators. Therefore, this study adopts a descriptive comparative framework, and no analytical equivalence or direct comparison of risk estimates between countries is assumed.

### 2.6. Ethical Aspects

This study did not require review by a Research Ethics Committee, as it exclusively used secondary databases, in the public domain and without nominal identification, as stipulated in the ethical regulations in force in Brazil and Portugal. In the Brazilian context, the study falls under CNS Resolution No. 510/2016, which waives ethical evaluation for research that uses publicly accessible information, duly anonymized and without the possibility of direct or indirect identification of individuals. Similarly, in Portugal, the use of data made available by the INE, also anonymized and publicly accessible, does not require submission to ethics committees or additional regulatory bodies. All procedures respected the principles of confidentiality, privacy, and responsible use of information, ensuring the methodological and ethical integrity of the research.

## 3. Results

Results are presented as parallel national analyses to allow contextual comparison of patterns and trends. Trends are presented across the 2020–2023 period, which coincides with the COVID-19 pandemic and its aftermath.

The analyzed sample of Brazilian women totaled 7689 women, predominantly of mixed race (52.5%), followed by white (31.7%), while indigenous (1.8%), Asian (0.3%), and unknown records (1.8%) represented minority proportions. Regarding age range, the highest concentration was observed in the 30–39 age group (45.2%), followed by 20–29 years (37.5%); extreme age groups showed low frequency, such as 10–14 years (0.5%) and 50–59 years (0.2%). As for regional distribution, the highest proportion of records occurred in the Northeast (30.5%) and Southeast (34.7%), while the North region accounted for 14.9%, the South for 10.6%, and the Central-West for 9.4%. Regarding education, almost half of the women had between 8 and 11 years of schooling (47.5%), followed by those with 12 or more years of schooling (15.2%). A total of 12% had their education classified as unknown, and only 1.7% had no formal education. Regarding marital status, the predominant condition was single (46.5%), followed by married (30%), while categories such as widow (0.4%) and legally separated (2.2%) showed low representation; 6.7% of the records were unknown (Table 1).

Analysis of 7689 maternal deaths revealed a well-defined pattern in the distribution of type, period, and causes of death. It was observed that the majority of deaths were classified as direct obstetric maternal deaths (50.6%), followed by indirect obstetric deaths (46.5%), while only 2.8% remained unspecified. Regarding the period of death, it was found that most deaths occurred in the postpartum period up to 42 days, representing 62% of all cases, followed by events during pregnancy, childbirth, or abortion (26.7%). Only a small proportion occurred in the late postpartum period (1.6%) or outside the pregnancy-puerperal cycle (1.8%). Inconsistent or missing records accounted for less than 8% of cases.

Maternal deaths were identified according to the International Classification of Diseases, 10th Revision (ICD-10), using codes O00–O99. For analytical purposes, causes were grouped into direct and indirect obstetric causes, following WHO recommendations. Direct obstetric causes included complications resulting from pregnancy, delivery, and the puerperium, particularly: O00–O08: pregnancy with abortive outcome, O10–O16: hypertensive disorders, O20–O29: other maternal disorders predominantly related to pregnancy, O30–O48: maternal care related to the fetus and amniotic cavity, O60–O75: complications of labor and delivery and O85–O92: complications predominantly related to the puerperium. Indirect obstetric causes corresponded to pre-existing or newly developed medical conditions aggravated by pregnancy, specifically: O98–O99: maternal infectious and non-infectious diseases complicating pregnancy, childbirth, and the puerperium. This classification guided the grouping of specific obstetric causes presented in Table 2, ensuring consistency between ICD-10 coding and analytical categories.

Regarding specific obstetric causes (ICD O00–O99), the main causes of death were other obstetric conditions not classified elsewhere, which accounted for 47.8% of deaths, followed by hypertensive disorders of pregnancy, childbirth and the puerperium (16.2%) and complications of labor (11.8%). Causes associated with the puerperium contributed to 9.5% of deaths, while pregnancies that ended in abortion accounted for 6.3%. Rare causes, such as complications directly classified as “childbirth” or behavioral syndromes/physical factors, accounted for less than 0.1%. According to the ICD-10 chapters, it was found that almost all maternal deaths (99.97%) were classified in chapter XV—Pregnancy, childbirth and the puerperium, confirming the obstetric nature of the events. Only two cases (0.03%) were recorded in Chapter V—Mental and Behavioral Disorders (Table 2).

The mixed-race category was used as a reference. It was observed that Black and Indigenous women presented substantially higher risks of maternal death, with IRRs of 1.636 and 1.644, respectively, both statistically significant (*p* < 0.001). These values reflect a mortality incidence almost 65% higher than that of mixed-race women. In contrast, white women presented a risk similar to the reference, while the yellow category showed a significantly lower risk, although with a low count of events. Maternal mortality increased sharply with advancing maternal age. Compared to the 20–29 year old group (reference), women aged 30–39 years had an IRR of 1.714 and women aged 40–49 years had an IRR of 2.716, both highly significant differences. These findings indicate that the incidence of maternal death may be up to 2.7 times higher at advanced maternal ages. Younger groups (10–14 and 15–19 years) showed lower IRRs, although the magnitude and interpretation of these values require caution, as they may reflect different exposure patterns or underreporting. Taking the Southeast region as a reference, regions with greater social vulnerability exhibited increased risks of maternal mortality. The North (IRR = 1.458) and Northeast (IRR = 1.207) regions showed significantly higher rates, confirming persistent regional inequalities. The South region presented a lower risk (IRR = 0.824), while the Central-West region exhibited a slightly higher risk (IRR = 1.162). These patterns reinforce the intersection between maternal mortality and geographic, socioeconomic, and health service access determinants (Table 3).

In Portugal, maternal mortality remained low throughout the study period, with small absolute numbers and year-to-year variability typical of low-incidence settings. Despite the limited availability of individual-level data, national reports indicate that the leading causes of maternal death are predominantly indirect obstetric causes, including pre-existing medical conditions aggravated by pregnancy, cardiovascular disease, and infections. Direct obstetric causes such as hemorrhage and hypertensive disorders occur less frequently but remain clinically relevant. These patterns reflect the epidemiological profile of high-income countries, where indirect causes increasingly predominate in maternal mortality.

Analysis of maternal mortality in Portugal between 2020 and 2023 shows an absolute decrease in the rate, although without statistical significance. In 2020, a year marked by the initial impact of the COVID-19 pandemic, the maternal mortality rate reached 20,1 deaths per 100,000 live births, the highest value in the series. In that year, it is estimated that approximately 17 maternal deaths occurred, with a wide confidence interval (95% CI: 10.5–29.7), reflecting greater statistical variability. In 2021, a significant reduction was observed, with a MMR of 8.8 per 100,000 live births (7 deaths). In 2022, there was a moderate increase to 13.1, followed again by a decrease in 2023, which recorded 10.5 deaths per 100,000 live births (9 deaths), with a confidence interval between 3.5 and 17.5.

The application of a Poisson regression model offset by the number of live births indicated an annual decreasing trend of approximately 14.2% (IRR = 0.8576); however, this trend did not reach statistical significance (*p* = 0.138), suggesting that, although maternal mortality fluctuated and globally decreased during the period, this variation can be attributed to the small absolute number of events and the natural variability of the estimates. Overall, the findings indicate that Portugal maintains relatively low levels of maternal mortality, with annual fluctuations reflecting both the small number of deaths and cyclical impacts, such as those observed in 2020. The lack of statistical significance in the trend reinforces the need for longer series or stratified data for a more robust assessment of temporal variations (Table 4).

## 4. Discussion

### 4.1. Overview and Summary of the Findings

The ecological nature of this study requires caution in interpretation. The associations observed cannot be interpreted as causal relationships. Important individual-level confounders such as socioeconomic status, comorbidities, and access to healthcare were not available in the Portuguese dataset and were only partially captured in Brazil. Therefore, the findings should be understood as indicators of population-level disparities rather than direct evidence of causal mechanisms. The comparison of causes between Brazil and Portugal must be interpreted cautiously due to differences in data granularity. While Brazilian microdata allowed detailed cause-specific analysis, Portuguese data were available only as aggregated national indicators. Nevertheless, a consistent pattern emerges: Brazil shows a higher burden of direct obstetric causes and pronounced sociodemographic inequalities, whereas Portugal reflects a profile typical of high-income countries, with a greater relative contribution of indirect causes. This contrast aligns with the global epidemiological transition in maternal mortality. Therefore, the binational comparison provides contextual insight rather than direct analytical equivalence, highlighting how different health system contexts and surveillance capacities shape maternal mortality profiles.

This binational observational study identified a contrasting and multifaceted picture of maternal mortality between Brazil and Portugal. Descriptively, Brazil presented persistently high rates, around 55–62 deaths per 100,000 live births in the analyzed period, while Portugal exhibited lower and fluctuating levels (8–20/100,000). This difference in magnitude between the countries is consistent with national analyses that point to a high and persistent level of maternal mortality in Brazil, especially in recent series that incorporate the pandemic period [1].

The observation of a predominance of direct obstetric deaths, with a substantial contribution from indirect causes, echoes global findings that document hemorrhage, hypertension, and increasingly, indirect causes (chronic diseases and infections) as relevant contributors to the burden of maternal mortality. Reviews and studies of global trends reinforce that, although hemorrhage still represents the largest single share, indirect causes have gained ground in the composition of maternal deaths, especially in contexts where pre-existing chronic conditions and infectious exposures (including COVID-19) overlap [10].

The heterogeneity observed by race, age, and region in the Brazilian population, with significantly higher IRRs among Black and Indigenous women, substantial risks in older age groups, and higher rates in the North and Northeast regions, is consistent with national studies that detail racial and territorial inequalities. Silva et al. (2024) [1] describe MMRs as almost double among Black women compared to white women in recent national series, highlighting that race remains a central determinant of maternal vulnerability in Brazil. These findings point to structural determinants (institutional racism, inequality in access to quality obstetric care, differences in the distribution of high-complexity services) and to multiple causal pathways that go beyond individual clinical factors [1].

The role of the COVID-19 pandemic is clearly evident in the temporal patterns of their study, with peaks in the early years of the pandemic and greater annual variability, and is supported by time series analyses that estimated increases/excesses in maternal mortality in Brazil during 2020–2021. Part of the increase observed in these years may reflect excesses directly attributable to COVID-19 and, in parallel, indirect effects (hospital overload, disruption of prenatal routines, delays in access to emergency obstetric care), which explain both the absolute increase and the regional variability. These findings are consistent with specific analyses of the pandemic period for Brazil [2].

From a methodological and interpretative point of view, two observations are central to explaining the consistency of their results with the literature: (1) rare events (maternal mortality) generate high statistical volatility, hence the usefulness of moving averages, age standardization and counting models with under/over dispersion checks; and (2) differences in the availability of microdata between Brazil (national microdata) and Portugal (aggregated series) limit the depth of binational inferences. The methodological literature and recent comparative studies highlight that modeled estimates and administrative data may diverge, especially in countries with few annual events, reinforcing the need for interpretative caution in direct comparisons [11,12].

Immediate implications from this synthesis: the findings of this study not only confirm pre-existing structural inequalities in Brazil, but also emphasize the need for multilevel policies that combine clinical interventions (management of hemorrhage, hypertension and obstetric emergencies), measures to control chronic diseases in the reproductive population, anti-racist strategies and strengthening of referral/counter-referral networks, especially in regions with less availability of high-complexity services. This recommendation is consistent with the literature that points to integrated actions as the most promising way to reduce maternal mortality in contexts of high inequality [13].

### 4.2. Direct and Indirect Impact of the COVID-19 Pandemic

Although temporal increases in maternal mortality were observed during the pandemic period, this study was not designed to estimate the direct effect of COVID-19. Therefore, the findings should be interpreted as temporal coexistence rather than causal attribution. Multiple concurrent factors, including health system disruption, changes in healthcare utilization, and broader social impacts, may have contributed to the observed patterns.

The COVID-19 pandemic impacted maternal mortality in two ways: (a) direct impact: Severe Acute Respiratory Syndrome Coronavirus 2 (SARS-CoV-2) infection in pregnant and postpartum women, which in many cases progressed to respiratory failure and death; and (b) indirect impact: interruption or degradation of access to reproductive health services (prenatal care, transportation to referral centers, emergency obstetric care), in addition to hospital overload that competed for resources and professionals. These mechanisms explain both the absolute increase and the greater regional variability observed in their study (peaks in 2020–2021 and differences between states/regions). Surveillance studies and time series in Brazilian contexts point to an excess of maternal deaths coinciding with periods of high transmission and system overload, corroborating the interpretation of direct and indirect effects on maternal mortality [14].

Hospital data and cohorts of pregnant women confirm that the clinical severity of COVID-19 in pregnancy was elevated at various points during the pandemic: infected pregnant women had higher rates of Intensive Care Unit (ICU) admission, need for mechanical ventilation, and death when compared to non-pregnant women of reproductive age, especially in the presence of comorbidities. Systematic reviews and multicenter studies highlight that, although much morbidity occurred among pregnant women with risk factors, there was also a considerable number of deaths in women without pre-existing comorbidities, suggesting failures in early recognition and adequate management. These findings support the hypothesis that some of the direct deaths from COVID-19 are attributable both to the severity of the disease and to gaps in obstetric care during the crisis [15].

National studies detailing pandemic waves in Brazil show abrupt increases in maternal deaths during epidemic peaks, with an accumulation of deaths in 2021 associated with more transmissible variants and periods of low vaccination coverage among pregnant women at that time. Brazilian clinical reports recorded hundreds of maternal deaths related to COVID-19 in 2020–2021 and documented differences in severity between regions, possibly linked to the unequal distribution of critical care beds and human resources [16,17].

In addition to deaths directly attributable to the infection, indirect effects were widespread: reduced prenatal visits, postponement of elective procedures, and logistical barriers to transporting pregnant women with signs of severity. Reviews on the impact of the pandemic on maternal services suggest a drop in demand for and supply of care, which translates into late diagnoses (pre-eclampsia, infections, hemorrhages) and worse obstetric outcomes. In scenarios with fragile services, this reduction in access can, in isolation, increase maternal mortality even when SARS-CoV-2 infection is not very prevalent [18].

The literature also draws attention to contextual and equity determinants that amplified the impact of the pandemic on more vulnerable women, rural populations, black women, women with lower education levels, and women residing in regions with low density of specialized care. Qualitative investigations and case analyses in Brazil have described a lack of support, delays in care, and signs of “obstetric racism” during the pandemic, factors that increase the risk of death even when the initial pathology did not seem inevitably fatal [19].

### 4.3. Racial Inequalities and the Persistence of Institutional Racism

The findings of this study, with an IRR of 1.64 for Black and Indigenous women compared to mixed-race women, confirm a consistent and alarming pattern of racial inequality in maternal mortality in Brazil. Maternal mortality among Black women has been systematically higher than among white and mixed-race women, with sharp peaks during the pandemic period, when the combination of social vulnerabilities and system overload amplified adverse outcomes [1].

Mechanisms that explain this excess risk are multi-interconnected. Reviews on racial disparities in maternal health point to the role of structural racism (unequal distribution of resources, lower investments in infrastructure in neighborhoods and regions predominantly inhabited by Black populations), implicit bias, and lack of cultural competence among professionals, which together result in lower quality of care, diagnostic delays, and undertreatment of risk signs [20].

In terms of lived experiences, investigations into “obstetric racism” and obstetric violence demonstrate that Black women frequently report disrespectful care, failure to recognize symptoms, and less clinical listening, factors that increase the risk of adverse outcomes [21]. Maternal mortality rates are much higher than those of the non-Indigenous population, and particular barriers are highlighted: long distances to referral maternity hospitals, transportation difficulties, an insufficient care network, and cultural communication problems between teams and traditional midwives. These logistical and cultural barriers explain why, in their data set and in related studies, Indigenous women present pronounced excess risks [22].

Racial inequalities interact with socioeconomic and territorial determinants: regions with less access to highly complex services and greater distances to referral centers (North and Northeast in their study) concentrate a higher proportion of deaths among Black and Indigenous women. The conjunction of race, poverty, and fragility of the network explains a substantial part of the regional variation in maternal mortality in Brazil, reinforcing that purely clinical policies are insufficient without structural transformations [23].

From a methodological and surveillance point of view, their results reinforce the urgent need for continuous monitoring with disaggregation by race, inclusion of socioeconomic variables, and qualitative investigation of preventable deaths. Only with data that cross-reference individual information, care context, and care trajectories will it be possible to identify breaking points (delays in seeking care, transportation, and attention) and design interventions that address institutional racism in clinical practice [24].

### 4.4. Maternal Age: Increasing Risks with Advancing Age

The findings of the present study, showing a progressive increase in the risk of maternal death with age (IRR ≈ 1.71 for 30–39 years and IRR ≈ 2.72 for 40–49 years, with extreme risk in the few observations of 50–59 years), are consistent with multiple recent evidence documenting a nearly linear relationship between advancing maternal age and worse maternal outcome. Although most pregnancies in older women have a favorable outcome, the likelihood of serious complications and maternal death increases with age, particularly from 35 to 40 years onwards, due to the accumulation of comorbidities and greater susceptibility to acute obstetric conditions [25].

The mechanisms that explain this risk gradient are multifactorial. Older women have a higher prevalence of chronic hypertension, diabetes, cardiovascular disease, and obesity, factors that increase the risk of severe pre-eclampsia, thromboembolic events, peripartum heart failure, and hemorrhagic complications, causes frequently associated with maternal death. Furthermore, age-related obstetric changes (e.g., placenta previa, placenta accreta, and more frequent obstetric procedures such as cesarean section) increase the likelihood of postpartum hemorrhage and the need for major interventions, contributing to the excess mortality observed in the older age groups [26].

Another relevant aspect is the interaction between age and access to quality care. In contexts with inequality in the provision of services (as evidenced regionally in the study), older women may accumulate biomedical vulnerabilities and barriers to timely care, amplifying the risk of death. In low- and middle-income countries, studies have shown that age extremes (adolescents and elderly women) present different degrees of risk that strongly depend on the ability of the health system to detect and manage complications early; that is, age alone is a risk marker that is modulated by system factors, organization of care, and comorbidities [27].

The results point to two priority actions. First, age-based risk stratification should be routinely incorporated into prenatal protocols, with more intensive surveillance (blood pressure monitoring, diabetes screening, cardiological evaluation when indicated) and birth planning in centers with obstetric support/maternal ICU for women ≥35–40 years with comorbidities. Second, public health interventions aimed at reducing chronic risk factors in the reproductive population (control of hypertension, diabetes and obesity, access to planned contraception and preconception counseling) can reduce susceptibility to serious outcomes in late pregnancies. Recent literature corroborates that such combined strategies can mitigate the increased risk observed in older age groups and decrease maternal mortality attributable to reproductive aging [25,26].

### 4.5. Regional Inequality and the Organization of the Health System

The findings of this study point to markedly higher maternal mortality rates in the North and Northeast regions compared to the South and Southeast, reflecting structural inequalities in the supply and organization of maternal care services in Brazil. The excess maternal deaths during the pandemic demonstrated heterogeneous regional trajectories and confirmed that regions with a lower density of high-complexity services and lower response capacity suffered proportionally greater increases in maternal mortality. This suggests that pre-existing weaknesses in the care network (insufficient obstetric/ICU beds, lack of specialized professionals and weak referral routes) amplified the impact of shocks such as COVID-19 [14].

The issue of geographic access is central to understanding the regional pattern. Recent studies that mapped hospital accessibility and distances traveled by pregnant women show that, in many municipalities in the North and rural areas, women need to travel long distances to access deliveries in hospitals with obstetric support and neonatal/maternal ICU, a plausible determinant of delays in care and worse outcomes. Geospatial evidence indicates that centralization policies without logistical support (transport, bed regulation, reception) increase the risk for women who live far from referral centers [28,29].

Beyond the geographic component, there is evidence that administrative organization and primary care programs directly influence maternal indicators. Recent evaluations of national programs indicate that changes in funding, the arrangement of primary care teams, and coordination between levels (primary care → intermediate → high complexity) impact the coverage and quality of prenatal care, risk screening, and timely referral [30]. The literature also documents that surveillance capacity and the use of administrative databases affect the detection and response to regional patterns. Regions with fragmented information systems or underreporting tend to underestimate the problem until a shock exposes the fragility of the network; on the other hand, better screening and monitoring (e.g., SIH–SIM–SINASC integration, surveillance panels) allow for the identification of critical points and the prioritization of interventions [23,31].

### 4.6. Profile of Causes: Balance Between Direct and Indirect Causes

In the present study, near-parity was observed between direct (50.6%) and indirect (46.5%) obstetric deaths, a pattern that requires careful interpretation in light of classifications, temporal changes (pandemic), and surveillance particularities. Conceptually, direct causes are those resulting from obstetric complications (hemorrhage, hypertension, puerperal infection, childbirth complications), while indirect causes refer to pre-existing or acquired conditions during pregnancy that are aggravated by gestation (heart disease, diabetes, anemia, chronic infections, COVID-19) [32].

Direct obstetric causes remain a central and largely preventable component of maternal mortality, particularly in middle-income settings [1,2,3]. The persistence of deaths related to hypertensive disorders, obstetric hemorrhage, sepsis, and complications of labor highlights critical gaps in the quality, timeliness, and coordination of maternal health care [1,2,3,4]. Compared with surveillance series and reports, the relative proportion of direct vs. indirect causes varies considerably by context and period. Reviews of maternal death reports indicate that in many countries, most deaths continue to be attributed to direct causes (hemorrhage, hypertension, infections), with a high average direct proportion in surveillance series (e.g., 70% direct in compilations of reports from 22 countries).

Hypertensive disorders of pregnancy continue to be one of the leading contributors to maternal death worldwide [2,5]. These conditions are highly preventable through early detection, risk stratification, adequate prenatal follow-up, and timely management of severe complications such as pre-eclampsia and eclampsia [5]. The persistence of these deaths suggests failures in the continuum of care, particularly in the transition from prenatal care to emergency obstetric services [3,6]. Obstetric hemorrhage remains another major cause of maternal mortality and represents a key indicator of the effectiveness of emergency obstetric care [2,7]. Most hemorrhage-related deaths can be prevented through timely recognition, availability of blood products, trained multidisciplinary teams, and adherence to standardized clinical protocols [7]. Strengthening obstetric emergency networks and ensuring rapid referral pathways are therefore essential priorities [3,7].

However, recent studies also document a proportional increase in indirect causes in several regions, especially those with greater epidemiological development and during the pandemic, when comorbidities and non-obstetric infections (including COVID-19) became prominent among the causes of maternal death [33,34]. Maternal sepsis and infection-related complications further reflect structural and organizational challenges within health systems, including delays in diagnosis, inadequate infection prevention, and barriers to timely treatment [4,8]. Improving hospital infection control, postpartum follow-up, and early recognition of warning signs should be central components of maternal health strategies [8].

Two main explanations help to understand why their study recorded such a high proportion of indirect causes. First, the impact of the COVID-19 pandemic abruptly increased deaths from indirect causes in 2020–2021: systemic SARS-CoV-2 infections, decompensation of chronic diseases, and disruptions in care (delays in diagnosis/treatment) increased the proportion of deaths classified as indirect in many contexts. Second, differences in the quality of classification and the availability of clinical information (for example, in locations with complete microdata, it is more feasible to accurately identify indirect causes) may alter the observed proportions between direct and indirect causes. These factors have already been documented in analyses showing an increased contribution of chronic diseases and aggravated conditions to the composition of maternal deaths in recent series [35,36].

From a clinical-programmatic point of view, the observed profile implies complementary responses: maintaining and improving interventions directed at direct causes (management protocols for obstetric hemorrhage, safety bundles for pre-eclampsia, control of puerperal infections) and simultaneously strengthening actions on indirect causes (detection and management of cardiovascular diseases, diabetes, anemia, screening and treatment of chronic infections; pre-conception integration and primary care) [37].

From a policy perspective, the persistence of preventable direct causes highlights the need to move beyond access to care toward quality of care and health system responsiveness [3,6]. Key priorities include strengthening emergency obstetric and neonatal care, implementing maternal early warning systems, and improving referral networks [6,7]. Importantly, many deaths associated with direct obstetric causes occur within health facilities, underscoring that increasing institutional birth coverage alone is insufficient; the focus must increasingly shift toward improving the quality, safety, and timeliness of care [3,6].

### 4.7. Portugal: Variability, Smaller Magnitude, and Data Limitations

The findings of the present study consistently lower, but fluctuating, Portuguese rates (8–20/100,000) should be interpreted in the context of a small absolute number of events, high sampling variability, and limitations in the availability of microdata. In countries with few annual maternal deaths, small absolute variations (for example, a difference of 5–15 deaths from one year to another) produce large changes in rates per 100,000 live births and wide confidence intervals, reducing the accuracy of temporal inferences and making it difficult to detect real trends without longer series [38].

In addition, the literature on comparative surveillance in European countries shows that differences in notification systems and classification methodologies (including underreporting and variations in the reclassification of causes) compromise direct comparisons between countries over time. Reliable monitoring of maternal mortality in low-frequency settings depends on additional procedures, e.g., clinical case reviews (maternal death reviews), linking death records and hospital records, and the use of multiple data sources, practices which, when inconsistent, lead to volatile and possibly biased estimates [39].

More recently, assessments that included the pandemic period highlighted a slight increase in 2020 followed by fluctuations in subsequent years, reflecting both the direct impact of COVID-19 and temporary changes in access to prenatal care and obstetric management. However, the interpretation of these signals in Portugal is limited by the lack of publicly available individualized microdata (e.g., detailed sociodemographic variables, causes coded by ICD-10 at the individual level), which prevents stratified analysis by age, origin, or comorbidities, analyses that in Brazil allowed the identification of disparities by race and region [40].

In short, the observations on Portugal in the present work, of low magnitude but with year-to-year variations, are consistent with the literature that warns of the inherent imprecision of short series with few deaths, the need for clinical review procedures and greater availability of microdata to allow stratifications and equity investigations. Practical recommendations include: (1) promoting the publication and controlled access to anonymized microdata when possible; (2) consolidating maternal death review programs; (3) monitoring vulnerable subgroups (immigrants, racialized populations) through mixed studies; and (4) interpreting annual trends with caution, preferring analyses in longer series or multi-year aggregations to reduce statistical volatility [38,39,40]. Thus, the comparison between countries should be interpreted as contextual and exploratory, highlighting convergences and divergences in patterns rather than establishing causal or risk equivalence. Thus, the value of this study extends beyond epidemiological description, offering a methodological reflection on the challenges of cross-national maternal mortality research.

### 4.8. Future Directions

Although the present study focuses on maternal mortality, the pathways leading to maternal death frequently originate from preventable postpartum complications. Therefore, future research and policy agendas should increasingly integrate epidemiological surveillance with emerging clinical strategies aimed at reducing maternal morbidity. Strengthening this bridge is essential to advance comprehensive maternal health care across the continuum from pregnancy to the postpartum period [1,2,3].

Maternal mortality represents the most severe outcome within a broader spectrum of maternal morbidity. Postpartum complications, including infection, hemorrhage, wound dehiscence, and delayed tissue repair, play a central role in the cascade that may ultimately lead to severe maternal outcomes. Consequently, innovations that improve postpartum recovery and reduce complications may indirectly contribute to lowering severe maternal morbidity and, in the long term, maternal mortality [1,4].

Recent clinical research has explored novel therapeutic approaches targeting wound healing in obstetric care. One emerging strategy involves the use of platelet-rich plasma (PRP) to enhance tissue repair following cesarean delivery. PRP is an autologous concentration of platelets rich in growth factors that promote angiogenesis, collagen synthesis, and tissue regeneration. Recent studies suggest that PRP application in cesarean scars may accelerate wound healing, reduce inflammation, and improve scar quality [5,41]. These findings indicate a promising adjunctive strategy to reduce postoperative complications such as infection and wound dehiscence, which remain important contributors to postpartum morbidity.

Another promising avenue involves lactic-acid–based formulations for episiotomy wound care. Lactic acid has demonstrated antimicrobial, anti-inflammatory, and tissue-regenerative properties. Emerging evidence indicates that topical lactic-acid formulations may improve episiotomy healing, reduce local inflammation, and shorten recovery time. Considering that episiotomy-related complications remain frequent in many obstetric settings, such innovations may represent an important strategy to improve postpartum recovery and maternal well-being [6,42].

Importantly, these approaches do not directly target maternal mortality; however, they address upstream determinants of severe maternal morbidity. The integration of clinical innovations aimed at improving postoperative recovery with population-level maternal health strategies may create synergistic effects across the continuum of care [2,3,4]. From a global health perspective, the translation of epidemiological evidence into clinical innovation represents a critical step toward strengthening maternal health systems. While public health interventions remain essential, including timely access to care, equitable health services, and improved surveillance systems, complementary clinical advances may help reduce complications that contribute to adverse maternal outcomes [1,2,3].

### 4.9. Limitations, Implications and Contributions to Clinical Practice

This study presents some methodological limitations that deserve consideration in the interpretation of the results. A major limitation of this study is the methodological asymmetry between countries. While Brazilian analyses were based on individual-level microdata enabling multivariable adjustment, the Portuguese component relied exclusively on aggregated national indicators. This difference restricts the control of confounding factors in Portugal, limits the comparability of risk estimates between countries, and reduces the depth of interpretation regarding sociodemographic inequalities. Another limitation is the absence of a formal statistical evaluation of the COVID-19 pandemic’s effect. Although the study period overlaps with the pandemic, no pre-post or interaction analyses were conducted. Consequently, interpretations regarding COVID-19 should be considered contextual and hypothesis-generating. Despite these limitations, the findings offer relevant implications for clinical practice and public policy planning. The identification of regional, racial, and age inequalities points to the need for a differentiated approach to obstetric care, with protocols that recognize the increased risk among Black, Indigenous, and older women. Clinical practice can benefit from the incorporation of active surveillance strategies during prenatal care, rigorous screening for comorbidities, and personalized birth plans according to risk profile. Strengthening primary health care, with effective articulation with secondary and tertiary levels, is essential to ensure that pregnant women with risk factors have rapid access to referral units and high-complexity care when necessary.

From a public health perspective, the results reinforce the importance of intersectoral policies that address the social determinants of maternal mortality, especially in more vulnerable regions. Expanding access to quality services, reducing geographical barriers, providing continuous training for healthcare teams, and implementing maternal death review programs are fundamental measures to improve surveillance and reduce preventable deaths. Furthermore, the findings contribute to the comparative literature between Brazil and Portugal by demonstrating that, although the epidemiological contexts and health systems are distinct, common challenges persist in monitoring maternal mortality and ensuring equity in obstetric care. Finally, this study contributes to the advancement of clinical practice by providing evidence that the integration of epidemiological surveillance and healthcare is crucial to reducing risks, improving maternal safety, and promoting reproductive justice in both countries analyzed.

## 5. Conclusions

In summary, the findings reinforce that reducing maternal mortality requires multi-strategic and intersectoral actions: strengthening primary care, expanding obstetric capacity in the most underserved regions, training teams sensitive to racial and socioeconomic inequalities, and adopting evidence-based maternal safety protocols. This binational analysis highlights persistent maternal mortality inequalities while underscoring major challenges in cross-country data comparability. The findings reinforce the urgent need for harmonized international maternal health surveillance systems capable of supporting robust global analyses and policy development. These findings should be understood within a descriptive comparative perspective, contributing to international dialog on maternal mortality and COVID-19 while underscoring the need for harmonized cross-national data to enable future analytical comparisons.

## Figures and Tables

**Table 1:** Absolute and relative distribution of sociodemographic profile characteristics of Brazilian women included in the study (n = 7689). Brazil 2020–2023.

Variable/Category	n	%
**Race**		
White	2436	31.7%
Black	917	11.9%
Yellow	21	0.3%
Brown	4036	52.5%
Indigenous	139	1.8%
Unknown	141	1.8%
**Age range**		
10–14 years	37	0.5%
15–19 years	600	7.8%
20–29 years	2880	37.5%
30–39 years	3473	45.2%
40–49 years	686	8.9%
50–59 years	14	0.2%
**Region of residence**		
North	1143	14.9%
North East	2343	30.5%
Southeast	2666	34.7%
South	813	10.6%
Central-West	725	9.4%
**Education**		
None	134	1.7%
1–3 years	435	5.7%
4–7 years	1380	17.9%
8–11 years	3650	47.5%
≥12 years	1169	15.2%
Ignored	922	12%
**Marital status**		
Single	3578	46.5%
Married	2309	30%
Widow	34	0.4%
Legally separated	166	2.2%
Other	1089	14.2%
Ignored	514	6.7%

**Table 2:** Classification of maternal deaths according to type, period and cause (n = 7689). Brazil 2020–2023.

Variable/Category	n	%	95% CI
**Type of maternal death**			
Direct obstetric	3892	50.6%	49.4–51.8
Indirect obstetric	3579	46.5%	45.3–47.7
Unspecified	218	2.8%	2.5–3.2
**Time of death**			
Pregnancy/childbirth/abortion	2054	26.7%	25.6–27.8
Postpartum up to 42 days	4768	62%	60.9–63.1
Postpartum 43 days–<1 year	122	1.6%	1.3–1.9
Not during pregnancy/postpartum	138	1.8%	1.5–2.1
Inconsistent period	3	0.04%	0.01–0.11
Not informed/ignored	605	7.9%	7.3–8.5
**Specific obstetric causes (ICD O00–O99)**			
Behavioral syndromes	2	0.03%	0.004–0.11
Pregnancy that ends in abortion	488	6.3%	5.8–6.9
Hypertensive disorders	1245	16.2%	15.4–1.7
Other pregnancy disorders	276	3.6%	3.2–4
Fetal/amniotic complications	361	4.7%	4.2–5.2
Labor complications	905	11.8%	11.1–12.6
Childbirth	1	0.01%	0.0002–0.07
Postpartum complications	730	9.5%	8.9–10.2
Other obstetric conditions	3682	47.8%	46.6–49
**ICD-10 Chapters**			
V—Mental/behavioral disorders	2	0.03%	0.004–0.11
XV—Pregnancy, childbirth and the postpartum period	7688	99.97%	99.94–99.99

**Table 3:** Maternal mortality by race, age group, and region. (n = 7689). Brazil 2020–2023.

	Deaths	Live Births (5 Years)	Rate 100,000	IRR	95% CI	*p*-Value
**Race**						
Brown (ref)	4036	706,8200	57.10	1.00	—	—
White	2436	420,0250	58.01	1.016	0.966–1.068	0.544
Black	917	981,625	93.45	1.636	1.523–1.758	<0.001
Yellow	21	59,780	35.12	0.615	0.401–0.945	0.026
Indigenous	139	148,115	93.85	1.644	1.388–1.946	<0.001
Ignored	141	229,910	61.33	1.074	0.908–1.270	0.404
**Age range**						
20–29 (ref)	2880	623,5505	46.19	1.00	—	—
10–14	37	69,695	53.07	0.707	0.517–0.967	0.030
15–19	600	144,6700	41.49	0.552	0.515–0.592	<0.001
30–39	3473	438,6710	79.19	1.714	1.599–1.837	<0.001
40–49	686	547,100	125.39	2.716	2.498–2.951	<0.001
50–59	14	1915	730.87	16.015	9.362–26.760	<0.001
**Region of residence**						
Southeast (ref)	2666	483,0800	55.20	1.00	—	—
North	1143	142,0985	80.45	1.458	1.360–1.562	<0.001
North East	2243	351,7240	66.66	1.207	1.142–1.276	<0.001
South	813	178,8060	45.47	0.824	0.762–0.891	<0.001
Central-West	725	113,0795	64.12	1.162	1.070–1.261	0.00034

**Table 4:** Maternal mortality rate per 100,000 live births. Portugal (2020–2023).

Year	Live Births	Deaths	TMM	95% CI
2020	84,371	16.96	20.1	10.5–29.7
2021	79,294	6.98	8.8	2.27–15.3
2022	82,632	10.82	13.1	5.30–20.9
2023	83,295	8.75	10.5	3.54–17.5

## Data Availability

These data were derived from the following resources available in the public domain: Sistema de Informação de Mortalidade (SIM) (http://tabnet.datasus.gov.br/cgi/tabcgi.exe?sim/cnv/mat10uf.def), Sistema de Informações sobre Nascidos Vivos (SINASC) (http://tabnet.datasus.gov.br/cgi/tabcgi.exe?sinasc/cnv/nvuf.def), Instituto Nacional de Estatística (INE) (https://www.ine.pt/xportal/xmain?xpid=INE&xpgid=ine_main) and Pordata from the Francisco Manuel dos Santos Foundation (https://www.pordata.pt/pt), accessed on 20 November 2025.

## Public Involvement Statement

No public involvement in any aspect of this research.

## Guidelines and Standards Statement

This manuscript was drafted against the Strengthening the Reporting of Observational Studies in Epidemiology (STROBE) [9].

## Abbreviations

ICU	Intensive Care Unit
INE	Instituto Nacional de Estatística
SARS-CoV2	Severe Acute Respiratory Syndrome Coronavirus 2
SDGs	Sustainable Development Goals
SIM	Sistema de Informação de Mortalidade
SINASC	Sistema de Informações sobre Nascidos Vivos
STROBE	Strengthening the Reporting of Observational Studies in Epidemiology

## References

[1] Silva A.D., Guida J.P.S., Santos D.S., Santiago S.M., Surita F.G. (2024). Racial disparities and maternal mortality in Brazil: Findings from a national database. Rev. Saude Publica.

[2] Cambou M.C., David H., Moucheraud C., Nielsen-Saines K., Comulada W.S., Macinko J. (2024). Time series analysis of comprehensive maternal deaths in Brazil during the COVID-19 pandemic. Sci. Rep..

[3] Carvalho-Sauer R., Costa M.D.C.N., Teixeira M.G., Flores-Ortiz R., Leal J.T.F.M., Saavedra R., Paixao E.S. (2024). Maternal and perinatal health indicators in Brazil over a decade: Assessing the impact of the COVID-19 pandemic and SARS-CoV-2 vaccination through interrupted time series analysis. Lancet Reg. Health Am..

[4] Ferreira N., Ferreira M., Santos E., Ferreira S., de Arriaga M.T., Costa A. (2025). Health literacy and its determinants among pregnant women in Portugal. BMC Public Health.

[5] Tavares M., Alexandre-Sousa P., Victória A., Loureiro S., Santos A.P., Mendes J. (2024). Experience of Labour and Childbirth in a Sample of Portuguese Women: A Cross-Sectional Study. Healthcare.

[6] Lee S., Kim S., Lee H., Park J., Son Y., López Sánchez G.F., Pizzol D., Lee J., Lee Y.J., Lee H. (2025). Global, Regional, and National Trends in Maternal Mortality Ratio Across 37 High Income Countries from 1990 to 2021, with Projections up to 2050: A Comprehensive Analysis from the WHO Mortality Database. J. Korean Med. Sci..

[7] Barros F.C., Gunier R.B., Rego A., Sentilhes L., Rauch S., Gandino S., Teji J.S., Thornton J.G., Kachikis A.B., Nieto R. (2024). Maternal vaccination against COVID-19 and neonatal outcomes during Omicron: INTERCOVID-2022 study. Am. J. Obs. Gynecol..

[8] Oliveira I.M.G.D., Fonseca E.P.D., França F.M.G., Cortellazzi K.L., Pardi V., Pereira A.C., Tagliaferro E.P.D.S. (2023). Age and Type of Delivery as Risk Indicators for Maternal Mortality. Rev. Bras. Ginecol. Obstet..

[9] von Elm E., Altman D.G., Egger M., Pocock S.J., Gøtzsche P.C., Vandenbroucke J.P., STROBE Initiative (2008). The Strengthening the Reporting of Observational Studies in Epidemiology (STROBE) statement: Guidelines for reporting observational studies. J. Clin. Epidemiol..

[10] Cresswell J.A., Alexander M., Chong M.Y.C., Link H.M., Pejchinovska M., Gazeley U., Ahmed S.M.A., Chou D., Moller A.B., Simpson D. (2025). Global and regional causes of maternal deaths 2009-20: A WHO systematic analysis. Lancet Glob. Health.

[11] Byun J.G., Lababidi S.M.D. (2026). National Trends in Maternal Mortality from Obstetric Causes in the United States, 2007–2023 [ID 2335]. Obstet. Gynecol..

[12] Ward Z.J., Atun R., King G., Sequeira Dmello B., Goldie S.J. (2025). Assessing differences in country-level estimates of maternal mortality: A comparison of GMatH, UN, and GBD model results for 2020. EClinicalMedicine.

[13] Souza J.P., Day L.T., Rezende-Gomes A.C., Zhang J., Mori R., Baguiya A., Jayaratne K., Osoti A., Vogel J.P., Campbell O. (2024). A global analysis of the determinants of maternal health and transitions in maternal mortality. Lancet Glob. Health.

[14] Orellana J.D.Y., Leventhal D.G.P., Flores-Quispe M.D.P., Marrero L., Jacques N., Morón-Duarte L.S., Boschi-Pinto C. (2024). Impact of the COVID-19 pandemic on excess maternal deaths in Brazil: A two-year assessment. PLoS ONE.

[15] La Verde M., Riemma G., Torella M., Cianci S., Savoia F., Licciardi F., Scida S., Morlando M., Colacurci N., De Franciscis P. (2021). Maternal death related to COVID-19: A systematic review and meta-analysis focused on maternal co-morbidities and clinical characteristics. Int. J. Gynaecol. Obstet..

[16] Cañedo M.C., Lopes T.I.B., Rossato L., Nunes I.B., Faccin I.D., Salomé T.M., Simionatto S. (2024). Impact of COVID-19 pandemic in the Brazilian maternal mortality ratio: A comparative analysis of Neural Networks Autoregression, Holt-Winters exponential smoothing, and Autoregressive Integrated Moving Average models. PLoS ONE.

[17] Scheler C.A., Discacciati M.G., Vale D.B., Lajos G.J., Surita F.G., Teixeira J.C. (2022). Maternal Deaths from COVID-19 in Brazil: Increase during the Second Wave of the Pandemic. Rev. Bras. Ginecol. Obs..

[18] Kassa Z.Y., Scarf V., Turkmani S., Fox D. (2024). Impact of COVID-19 on Maternal Health Service Uptake and Perinatal Outcomes in Sub-Saharan Africa: A Systematic Review. Int. J. Environ. Res. Public Health.

[19] Diniz D., Brito L., Rondon G. (2022). Maternal mortality and the lack of women-centered care in Brazil during COVID-19: Preliminary findings of a qualitative study. Lancet Reg. Health Am..

[20] Montalmant K.E., Ettinger A.K. (2024). The Racial Disparities in Maternal Mortality and Impact of Structural Racism and Implicit Racial Bias on Pregnant Black Women: A Review of the Literature. J. Racial Ethn. Health Disparities.

[21] Santana A.T., Couto T.M., Lima K.T.R.D.S., Oliveira P.S., Bomfim A.N.A., Almeida L.C.G., Rusmando L.C.S. (2024). Obstetric racism, a debate under construction in Brazil: Perceptions of black women on obstetric violence. Cien. Saude Colet..

[22] Ferreira G.A.T., Cherchiglia M.L., Valk M., Pilecco F.B. (2025). Mortality trends among Indigenous women of reproductive age in Brazil: A time series analysis. Lancet Reg. Health Am..

[23] Tenorio D.S., de Matos Brasil A.G., Nogueira B.G., Rolim Lima N.N., Araújo J.E.B., Rolim Neto M.L. (2022). High maternal mortality rates in Brazil: Inequalities and the struggle for justice. Lancet Reg. Health Am..

[24] Hailu E.M., Maddali S.R., Snowden J.M., Carmichael S.L., Mujahid M.S. (2022). Structural racism and adverse maternal health outcomes: A systematic review. Health Place.

[25] Tanaka H., Hasegawa J., Katsuragi S., Tanaka K., Arakaki T., Nakamura M., Hayata E., Nakata M., Murakoshi T., Sekizawa A. (2023). High maternal mortality rate associated with advanced maternal age in Japan. Sci. Rep..

[26] Hochler H., Lipschuetz M., Suissa-Cohen Y., Weiss A., Sela H.Y., Yagel S., Rosenbloom J.I., Grisaru-Granovsky S., Rottenstreich M. (2023). The Impact of Advanced Maternal Age on Pregnancy Outcomes: A Retrospective Multicenter Study. J. Clin. Med..

[27] Nyongesa P., Ekhaguere O.A., Marete I., Tenge C., Kemoi M., Bann C.M., Bucher S.L., Patel A.B., Hibberd P.L., Naqvi F. (2023). Maternal age extremes and adverse pregnancy outcomes in low-resourced settings. Front. Glob. Womens Health.

[28] Pinho Neto V., Machado C., Lima F., Roman S., Dutra G. (2024). Inequalities in the geographic access to delivery services in Brazil. BMC Health Serv. Res..

[29] Albuquerque P.C., Almeida L.M., Sá L.M.A., Silva L.L., Melo T.F., Camargo K.R. (2025). Geographic accessibility to hospital childbirths in Brazil (2010–2011 and 2018–2019): A cross-sectional study. Lancet Reg. Health Am..

[30] Domingues R.M.S.M., Rodrigues A.S., Dias M.A.B., Saraceni V., Francisco R.P.V., Pinheiro R.S., Coeli C.M. (2024). Maternal health surveillance panel: A tool for expanding epidemiological surveillance of women’s health and its determinants. Rev. Bras. Epidemiol..

[31] Ranzani O.T., Marinho M.F., Bierrenbach A.L. (2023). Usefulness of the Hospital Information System for maternal mortality surveillance in Brazil. Rev. Bras. Epidemiol..

[32] Alipour J., Payandeh A., Karimi A. (2023). Prevalence of maternal mortality causes based on ICD-MM: A systematic review and meta-analysis. BMC Pregnancy Childbirth.

[33] Muriithi F.G., Ameh C.A., Gakuo R.W., Williams C.R., Devall A., Coomarasamy A., Fawcus S. (2025). Variability in the Causes and Delay Factors Contributing to Maternal Mortality: Evidence from Maternal Death Surveillance Reports of 22 African Countries. BJOG.

[34] Jiang J., Qin L., Wang B. (2025). Long-term dynamic burden and attributable risk analysis of indirect maternal mortality over the past 30 years: An observational study and insights for perioperative management in the next decade. Int. J. Surg..

[35] Sabr Y., Lisonkova S., Mayer C., Joseph K.S. (2025). Temporal Changes in the Contribution of Chronic Disease to Maternal Mortality in the United States. Paediatr. Perinat. Epidemiol..

[36] Wu N., Ye E., Ba Y., Caikai S., Ba B., Li L., Zhu Q. (2024). The global burden of maternal disorders attributable to iron deficiency related sub-disorders in 204 countries and territories: An analysis for the Global Burden of Disease study. Front. Public Health.

[37] Ekwuazi E.K., Chigbu C.O., Ngene N.C. (2023). Reducing maternal mortality in low- and middle-income countries. Case Rep. Womens Health.

[38] Gomes M.C., Ventura M.T., Nunes R.S. (2012). How many maternal deaths are there in Portugal?. J. Matern. Fetal Neonatal Med..

[39] Bouvier-Colle M.H., Mohangoo A.D., Gissler M., Novak-Antolic Z., Vutuc C., Szamotulska K., Zeitlin J., Euro-Peristat Scientific Committee (2012). What about the mothers? An analysis of maternal mortality and morbidity in perinatal health surveillance systems in Europe. BJOG.

[40] Peppe M.V., Costa F.B.P.D., Pedrilio L.S., Prado C.A.C., Baraldi A.C.P., Stefanello J. (2025). Analysis of the influence of the COVID-19 pandemic on maternal mortality trends, 2011–2021. Rev. Lat. Am. Enferm..

[41] Brezeanu A.-M., Brezeanu D., Tica V.-I. (2025). Intraoperative Platelet-Rich Plasma (PRP) for Post-Cesarean Scar Healing: A Single-Center Randomized Controlled Pilot Study. Healthcare.

[42] Brezeanu D., Brezeanu A.-M., Tica V. (2026). Lactic Acid-Loaded Hydrogels for Post-Episiotomy Wound Healing: Microenvironment Engineering and Regenerative Strategies—A Narrative Review. Molecules.

